# Effectiveness of a Digital Health Application for the Treatment of Diabetes Type II—A Pilot Study

**DOI:** 10.3390/jcm12196317

**Published:** 2023-09-30

**Authors:** Maxi Pia Bretschneider, Lena Roth, Peter E. H. Schwarz

**Affiliations:** 1 Department for Prevention and Care of Diabetes, Department of Medicine III, Faculty of Medicine Carl Gustav Carus, Technische Universität Dresden, 01307 Dresden, Germany; maxi.bretschneider@mailbox.tu-dresden.de (M.P.B.); peter.schwarz@ukdd.de (P.E.H.S.); 2 Paul Langerhans Institute Dresden, Faculty of Medicine, Technische Universität Dresden, 01307 Dresden, Germany

**Keywords:** diabetes mellitus type 2, self-management, digital health, HbA1c, lifestyle intervention, digital intervention, mHealth

## Abstract

(1) Background: This study aimed at providing preliminary evidence for mebix, an app-based treatment program for patients with diabetes mellitus type II. The main target was to show a positive healthcare impact as defined by improved blood glucose control, i.e., reduced HbA1c values. (2) Methods: For this, a 3-month, prospective, open-label trial with an intraindividual control group was conducted. Participants received the mebix intervention for 3 months. HbA1c values were observed every 3 months: retrospectively, at baseline, and 3 months after the start of using the app. Additionally, weight and patients’ reported outcomes (well-being, diabetes-related distress, and self-management) were assessed. Data generated within the app were summarized and analyzed (steps, physical activity, fulfilled tasks, and food logs). (3) Results: After the usage of mebix for 3 months, participants significantly reduced their HbA1c levels (−1.0 ± 0.8%). Moreover, improvements in weight, well-being, and self-management as well as a reduction in diabetes-related distress were observed. App-generated data mainly supported the other main finding, that higher baseline HbA1c values lead to higher reductions. Overall, the study provided preliminary evidence that mebix can help patients improve metabolic and psychological health outcomes.

## 1. Introduction

Currently, it is estimated that 537 million people (20 to 79 years old) worldwide live with diabetes mellitus, and the prevalence is expected to increase further [1]. The primary form affecting 90% of diabetes mellitus patients is type II diabetes mellitus (T2DM) [2]. The prevalence in Germany is estimated to be between 7.2% and 9.9% and increases steadily with age, reaching a prevalence of over 30% (age group 75–79) [3,4].

T2DM is a metabolic disease characterized by hyperglycemia, i.e., chronically elevated blood glucose levels caused by impaired insulin production and insulin resistance. The standard measure to diagnose T2DM is an HbA1c value above 6.5% [5,6]. 

Guidelines for the treatment of T2DM define improved glycemic control as the target, expressed as HbA1c values between 6.5% and 7.5% depending on the individual disease burden, additional comorbidities, and pharmacotherapies of the patient [6,7]. Since T2DM is strongly associated with overweight, obesity, and elevated blood pressure as well as blood lipids, the therapy also aims at decreasing weight, elevated blood pressure, and blood lipids to reduce increased mortality cardiovascular risks and other health complications associated with T2DM [8,9,10]. 

Independently of possible pharmacotherapies, the basis of T2DM therapy are multimodal interventions that should help patients develop a healthier lifestyle and maintain psychological well-being [8]. This includes support and education for diabetes self-management, the development of a healthy diet, increased physical activity, weight management, and smoking cessation [5,7,8]. Apart from physiological difficulties and comorbidities, T2DM patients often suffer from disease-related distress causing impairments in quality of life and well-being up to depression. Being mutually dependent, improving self-management and psychological well-being are relevant aspects of T2DM behavioral interventions [11]. 

Diabetes technologies are an integral part of treatment, including insulin pumps or allowing continuous monitoring of blood glucose [10]. Moreover, technology is used to enhance and provide support and treatment for diabetes patients [10]. Another field that is evolving is digital medical devices as (stand-alone) interventions for chronic diseases like diabetes combining self-monitoring and education [12]. Two meta-analyses looking at the potential of telemedicine and (stand-alone) app-based interventions showed the potential of such in glycemic control and a reduction in HbA1c values [13,14]. However, both meta-analyses concluded that evidence is limited. 

Since 2019, Germany has introduced the possibility that digital health applications are fully covered by health insurances after they are approved by the German Federal Institute for Drugs and Medical Devices (BfArM). Approval is only granted after verification of a positive healthcare effect and fulfillment of quality, data security, and risk requirements [15]. 

This study was conducted to show the preliminary evidence according to § 139e SGB V of mebix (Vision2B GmbH, Erfurt, Germany), an app-based treatment program for patients with T2DM. Thus, the primary objective is to determine if the use of mebix is associated with an impact on glycemic management measured as a change in HbA1c in patients with T2DM. 

## 2. Materials and Methods

### 2.1. Study Design

**Figure 1:**
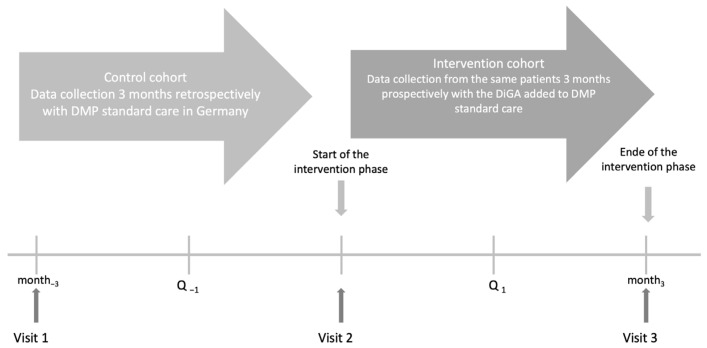
Study Design. Note: DiGA, Digital Health Intervention; DMP, Disease Management Program.

To evaluate the effect of mebix on HbA1c in patients with T2DM an EDDY study design was applied (evaluation of the impact of DiGAs in Patients with type 2 diabetes mellitus in daily practice). The EDDY trial design consists of a 3-month, prospective, multicenter, open-label observational study with an intraindividual control group (see Figure 1) and was already employed and described previously [16]. The study is registered in the German Clinical Trials Registry (DRKS00032547) and was approved by the Ethics Committee at the Technical University of Dresden (BO-EK-195032021) on 7 May 2021.

### 2.2. Recruitment and Study Procedures

Type 2 diabetes patients older than 18 years, with a HbA1c between 7.5% and 11%, and who are able and willing to use mebix, were included in the study. Exclusion criteria included: using other apps for diabetes management, participation in a weight loss program in the last 6 months, an insulin pump or continuous glucose monitoring, impairments—including mental or psychic impairments—or conditions which, in the opinion of the investigator, would seriously compromise the integrity of the study.

Participants were recruited online via social media campaigns. The potential participants were guided to the study website where they were asked to fill in an eligibility criteria questionnaire and sign an informed consent form. The patients who met the eligibility criteria and gave their informed consent were asked to book an onboarding call with the mebix study advisor. During the call, the mebix study advisor confirmed the patient’s eligibility and introduced the study procedures. The enrolled participants received a package with study information materials, electronic patient-reported outcomes forms as well as information material for the physician, and a standardized observational study case report form (CRF). 

The CRF was used for the collection of HbA1c values at 3 time points (3 months before the start of the use of mebix, baseline at the start of using mebix, and 3 months after the start of using mebix). This information was filled out and signed by the physician, a member of the care team, or the patient himself or herself. Furthermore, electronic patient-reported outcome questionnaires were used. These questionnaires were not provided within the app but were sent to the participants in electronic form online at 3 time points (baseline at the start of mebix, optionally at 6 weeks after starting to use mebix, and 3 months after starting to use mebix). A participant was considered to have completed the study if all phases of the study, including the final visit 3 months after starting to use mebix, had been completed. Participants received a financial reward of EUR 30 in the form of an Amazon Voucher when all study data were delivered (i.e., HbA1c at all 3 time points and patient-reported outcomes at baseline and 3 months). Participants withdrawing their consent did not proceed to the final visit.

### 2.3. Intervention

The intervention group used the mebix app with all functionalities for 3 months in addition to standard diabetes care defined in the diabetes disease management program provided by their diabetes specialist and/or general practitioner.

mebix is a certified Class I medical device under the Medical Device Regulation 2017/745. As an app-based therapy, it is designed to improve T2DM control by enabling patients to adopt better self-management and lifestyle decisions, leading to an improved state of health and quality of life. It complements pharmacological or other therapy initiated by a physician and supports patients in reaching their treatment goals. 

mebix employs a guideline-based, multimodal therapy approach to provide individualized support in lifestyle modification and self-management. It consists of a 90-day app-guided program using a system of goals, daily tasks, and automated messages, as well as educational content, thus serving as a continuous toolkit. The interactive educational course covers topics across motivation, goal setting, diet and personalized nutrition, physical activity, mental well-being, and social aspects of life with diabetes. The teaching is implemented using relevant personal goals that help patients root important habits into their daily lives. Patients are also engaged by a system of motivational and educational micro-messages, which help nudge them to learn more about their physiological and lifestyle parameters. To achieve individual lifestyle goals, several tracking tools for diet, physical activity (either manually or automatically in the form of step data from the Google Fit or Apple Health App), and medication allow participants to monitor their behavior. Through a diary, patients can also track several physiological parameters like their blood glucose, blood pressure, or weight.

In the beginning, a special focus lay on diet and 12-week personalized guidance aiming to help optimize blood glucose responses to food. It consists of dietary habit tracking via a nutrition database with automated feedback in the form of a traffic light system. 

Overall, the app is designed for minimum manual logging and optimal usability for older generations. To ensure patient safety and enhance the effective use of the program, a chat advisor is available on chat to answer patients’ questions. Examples of the user interface of the mebix app can be found in the Supplementary Materials. 

### 2.4. Comparator

The control group consisted of retrospective observations of the same subjects that were in the intervention group as the study is based on an intraindividual retrospective design. The control group received standard diabetes care defined in the diabetes disease management program provided by their diabetes specialist and/or general practitioner.

### 2.5. Outcome Measurement

The primary objective was to determine if the use of mebix is associated with a clinically meaningful impact on glycemic management in the change in HbA1c in diabetic patients, i.e., the change in HbA1c after 3 months of using mebix compared with an intraindividual control group. In reference to other studies, a clinically meaningful outcome was defined as an HbA1c difference of 0.5 HbA1c % points [13,16]. The secondary objective was to determine if the use of mebix is associated with changes in body weight and BMI. To evaluate changes in well-being, depression, and self-management validated patient-reported outcome (PRO) measurements were collected. Changes were assessed with the following questionnaires: for self-management, the Summary of Diabetes Self-Care Activities measure (SDSCA) [17,18]; for well-being, the WHO-Five Well-Being Index (WHO-5); and for depression, the short version of the Problem Areas in Diabetes questionnaire (PAID-5) that assesses emotional distress caused by the disease but can also be used as a screening tool for depression [19,20]. Generally, to detect potential impairments in well-being and increased diabetes-related distress cut-off values of 12 for the WHO-5 [21] and 8 for the PAID-5 are suggested [19]. 

Additional app-reported and generated data were used to assess therapy adherence, i.e., usage of mebix, predefined as at least 3 days of nutritional tracking and weekly usage, as well as their impact on lifestyle (average step count and minutes of physical activity). 

### 2.6. Sample Size

An a priori power analysis was performed using G*Power [22,23]. Based on a recent meta-analysis the expected change in HbA1c of 0.5% represents an effect size of 0.5 with SD (standard deviation) of 1% [13]. Considering a two-tailed *t*-test with a power and alpha of 80% and 5%, respectively, the number of participants needed to detect the difference is 34. Based on earlier studies with the same design, a dropout rate of 40% was assumed, leading to a sample size of 57.

### 2.7. Statistical Analysis

Patients recorded the HbA1c value 3 months prior to the beginning of the mebix program, in the meantime, continuing their treatment via national standards of care. The second value was recorded at the beginning of the mebix journey, and the final outcome was recorded 3 months into the program. Repeated measures ANOVA with Bonferroni correction was used to assess the overall significance of the difference in HbA1c values. The difference in outcomes between mebix and current standards of care was obtained by running several post hoc tests. Missing HbA1c values from the retrospective control group were replaced by the mean value of all the remaining patient records. The aim was not to generate any additional benefit in favor of the intervention through missing values. 

The change in secondary outcomes, i.e., body weight, BMI, and PRO, were evaluated using paired *t*-tests to compare the baseline and 3-month follow-up values. Missing data at baseline or 3 months were replaced by the 6-week follow-up questionnaire. 

## 3. Results

### 3.1. Participant Characteristics

**Figure 2:**
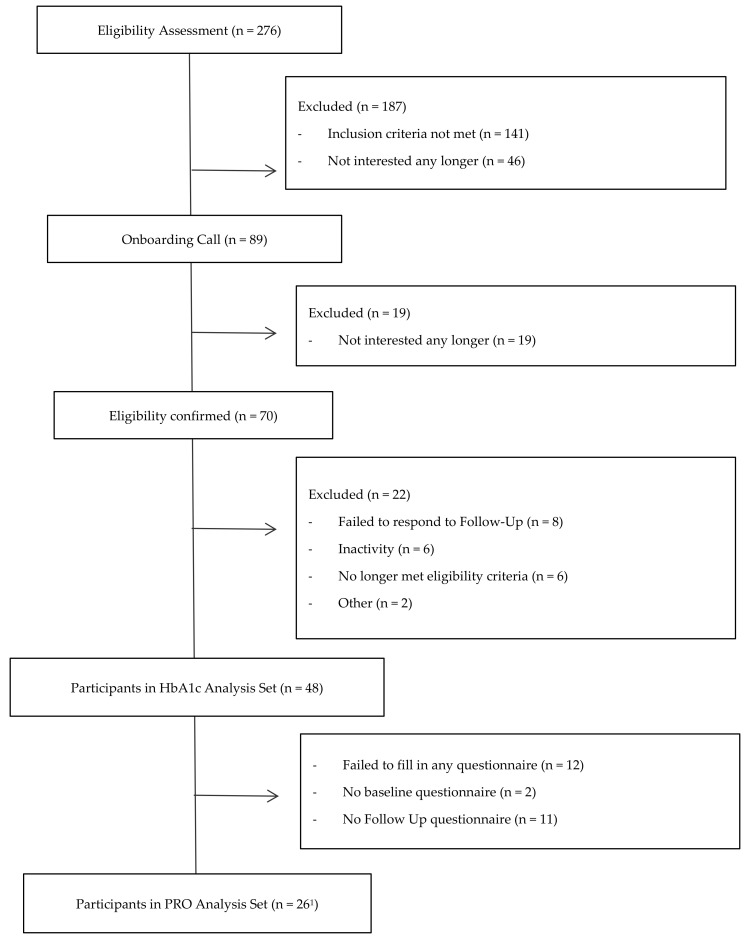
Participant Flow Chart. Note: PRO, Patient-Reported Outcomes. ^1^ The 26 participants were included in the PRO Analysis Set since both a baseline and follow-up questionnaire (after 3 months) had to be available. Missing questionnaires were partly imputed with an interim questionnaire at 6 weeks.

The entire process of eligibility assessment and enrolment in the study is illustrated in Figure 2. A total of 70 participants were found to be eligible and enrolled in the study. For the analysis, 22 participants were excluded because they no longer met the inclusion criteria (i.e., HbA1c values outside of the accepted range of 11%, n = 6), or because they did not respond to the follow-up, and hence no measurement points were available (n = 8); or due to inactivity in the app (n = 6) or other reasons (n = 2). Of the remaining 48 participants, all three HbA1c values were available, hence no HbA1c value was imputed for the analysis. Baseline and 3-month follow-up questionnaires, including the PRO, were available for 19 (39.6%) patients. The six-week questionnaire was imputed twice for baseline and follow-up, and three times for both baseline and follow-up. As a result, 37 (77.1%) baseline and 28 (58.3%) follow-up questionnaires were available. To perform a repeated measures ANOVA, however, after imputation, baseline and follow-up questionnaires were available for 26 (54.2%) patients. On average participants were 58.8 ± 9.0 years old, and 50% were male.

### 3.2. Effects on Glycemic Control

The mean HbA1c baseline value for the intraindividual control group (i.e., retrospective data from 3 months before the start of using mebix) was 8.4 ± 0.9% and decreased on average by 0.2 ± 0.8%, leading to a follow-up value of 8.3 ± 0.7% (i.e., the baseline value of the intervention group) (see Table 1). After the 3 months of using mebix, the mean HbA1c value decreased on average by 1.0 ± 0.8%, resulting in a 3-month follow-up value of 7.3 ± 0.6% (see Table 1). 

### 3.3. Summary of the Results of Glycemic and Metabolic Parameters

The repeated measures ANOVA revealed significant differences between the time points [F (2, 94) = 54.47, *p* < 0.001]. Paired *t*-tests with Bonferroni correction revealed a non-significant decrease for the control period (*p* = 0.177) and a significant decrease for the intervention period (*p* < 0.001). Additionally, the mean changes were significantly higher in the intervention period compared to the control period (*p* < 0.001). Overall, the results suggest that the treatment with mebix achieves a clinically meaningful effect on lowering HbA1c, which is also superior when compared to standard diabetes treatment. 

Retrospectively 3 months as well as at baseline before the start of using mebix one of the 48 participants (2.1%) had an HbA1c value below the recommended range of 7%. After 3 months of the intervention, 19 participants (39.6%) had HbA1c values below the recommended range. 

**Figure 3:**
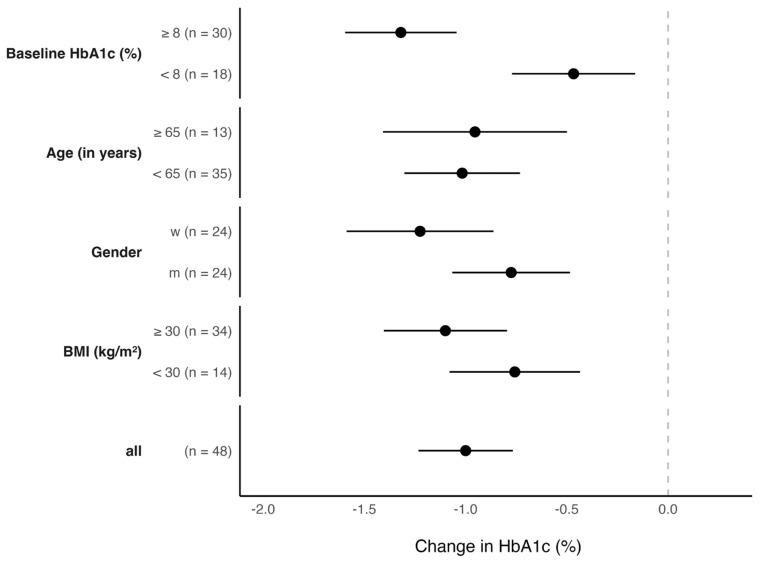
Results of Glycemic Control in the intervention group overall and by subgroup after 3 months of using mebix. Shown are the mean changes in HbA1c and the 95% confidence intervals.

In addition, the explorative analysis examined subgroup differences in changes in HbA1c values within the intervention group, i.e., baseline HbA1c (</≥8%), age (</≥65 years), gender (m/w), and baseline BMI (</≥30 kg/m^2^). The subgroup analysis showed that older participants reduced their HbA1c values slightly less (−1.0 ± 0.8%) than younger participants (−1.0 ± 0.8%); that women reduced their HbA1c values more (−1.2 ± 0.9) than men (−0.8 ± 0.7%); participants with a higher baseline BMI reduced their HbA1c values more (−1.1 ± 0.9%) than participants with a lower baseline BMI (−0.8 ± 0.6%), and that a higher baseline HbA1c value is associated with a higher reduction (−1.3 ± 0.7%) compared to a lower baseline HbA1c (−0.5 ± 0.6%). The two-sided *t*-test, after Bonferroni correction, revealed a significant difference between high/low baseline HbA1c values; all other subgroups did not yield significance. The results are summarized in Figure 3. 

### 3.4. Effects on Metabolic Parameters

As an additional metabolic parameter, patients reported their weight before the start of the intervention and after 3 months. Of the 48 participants, 39 (81.3%) weight measurements were available. At baseline, the average weight of the participants was 101 ± 18.1 kg, corresponding to a baseline BMI of 35 ± 6.5 kg/m^2^. Most patients had obesity grade I (n = 17, 35.4%), followed by obesity grade II and III (each n = 8, 16.7%) and overweight (n = 6, 12.5%). On average the weight was reduced by 2.9 ± 3.4 kg (−2.8 ± 3.0%) at the 3-month follow-up, leading to an average weight of 98.2 ± 17.7 kg and corresponding to a follow-up BMI of 34 ± 6.5 kg/m^2^ and a BMI reduction of 1 ± 1.1 kg/m^2^ (see Table 1). These reductions in weight (in kg) and BMI were significant (*p* < 0.001). After the intervention, most participants still had obesity grade I (n = 14, 29.2%), followed, however, by overweight (n = 9, 18.8%), grade III obesity (n = 8, 16.7%), and grade II obesity (n = 6, 12.5%). 

### 3.5. Effects on Patient-Reported Outcomes

PRO, i.e., well-being, changes in diabetes-related emotional distress, and self-management were only reported by the intervention group. The summary of baseline and follow-up results are shown in Table 2. 

To evaluate changes in PRO, only participants with both baseline and 3-month follow-up questionnaires were included in the analysis (n = 26). 

After 3 months, well-being increased by 1.1 ± 4.0, and diabetes-related emotional distress decreased by 0.8 ± 2. At baseline, 13 patients overcame the cut-off of 3 for the WHO-5 and 8 for the PAID-5, suggesting decreased well-being and increased diabetes-related distress, respectively. This number was reduced to 0 for the WHO-5 and 8 for the PAID-5 after 3 months. Self-management increased by 0.4 ± 0.9. Looking at the subgroups of self-management, footcare improved the most by 1.3 ± 1.5, followed by nutrition by 0.2 ± 1.2 and activity by 0.3 ± 1.3 while blood sugar control only slightly improved by 0.1 ± 1.7. After Bonferroni correction for self-management, only footcare increased significantly. 

Two questions of the SDSCA questionnaire regarded smoking. Two of the 26 analyzed participants who had smoked at baseline smoked the same amount at the 3-month follow-up (25 and 30 cigarettes per day). One participant started smoking (20 cigarettes per day). All other participants were non-smokers. 

### 3.6. App-Reported Data

Available app data give insights into the physical activity by average daily steps and tracked time of additional physical activity as well as food tracking (rather, whether the recommended 3 days of food tracking were completed) and general app usage (i.e., if all the 12 weekly tasks were completed). 

Of the 48 analyzed participants, 27 (56.3%) completed all weekly tasks, and on average 9.5 ± 3.5 tasks were completed. For 32 (77.8%) participants, food tracking information was available, and 26 of the 48 participants (54.2%) completed at least three days of food tracking; on average 2.8 ± 0.5 days were completed. Information on physical activity was available for 27 of the 48 participants (56.2%). The average number of daily steps was 5250 ± 2160, with 14 of the 48 participants (29.2%) having an average daily step count above 5000. On average 297 ± 240 min of physical activity were tracked per week, corresponding to 4.6 ± 4 h a week. 

To understand the effect of mebix on glycemic control, the Pearson’s correlation coefficient was calculated between the change of HbA1c (%) from baseline to 3-month follow-up and the numeric variables number of weekly tasks completed, number of food logs, average daily steps, and weekly tracked physical activity (in minutes). The number of completed daily tasks showed no correlation (r = 0.0), and the average daily steps (r = 0.2) and the number of food logs (r = 0.3) showed a low positive correlation indicating that with a higher daily step count and more food logs, the HbA1c reduction might be slightly less pronounced. Physical activity (in minutes) was slightly negatively correlated with the change in HbA1c (r = −0.2) indicating that more physical activity led to a slightly more pronounced HbA1c reduction. Interestingly, all the app interactions showed none to low correlations indicating that adherence to usage might differ between different app features. 

To analyze the explained variance in HbA1c change with the app data, a multiple linear regression model was fitted with a change in HbA1c (in %) from baseline to follow-up depending on the variable. Due to the small variance in the number of daily tasks completed and the number of food logs, both variables were included as factorial predictors and coded as to whether or not the participants fulfilled the protocol (i.e., all 12 weekly tasks and completed; food was tracked on at least 3 days). The average daily steps and weekly tracked physical activity (in minutes) were included as numeric predictors. Based on the results of the subgroup analysis that showed a significant difference in HbA1c change based on a high or low HbA1c value (in %), the baseline HbA1c (in %) was added as a covariate. Notice that before fitting the model all predictors were tested for correlation to prevent multicollinearity in the model. Overall, the model showed a good fit (Adjusted R^2^ = 0.82). The results of the ANOVA are shown in Table 3. 

The results show that the average daily steps, whether or not 3 food logs were completed, and the baseline HbA1c baseline value significantly explain variance in HbA1c changes from baseline to 3 months follow-up in the intervention group. Looking at the coefficients of the model, the relationship between HbA1c change and daily average steps is positive while HbA1c change and baseline HbA1c are negatively related. This suggests that with a higher average daily step count, the HbA1c reduction is less pronounced, while the reduction is more pronounced the higher the baseline HbA1c value. 

## 4. Discussion

The present study investigated the preliminary effectiveness of the digital lifestyle intervention mebix on glycemic control, weight as well as self-management, well-being, and diabetes-related emotional distress. After the intervention, no patient overcame the cut-off for impaired well-being, and the number of patients with increased diabetes-related distress was reduced.

The results show that HbA1c values significantly improved after 3 months of using mebix, i.e., an average reduction of 1.0 ± 0.8% was achieved. This reduction was also significantly higher compared to the control period in which participants did not use the app and on average reduced their HbA1c by 0.2 ± 0.8%. In the control period, the number of participants who had an HbA1c value within the guideline-recommended target range of below 7.0% remained constant [7,24]. After the 3-month intervention, the number of participants who achieved HbA1c values below 7% increased from 2.1% to 39.6%. Moreover, secondary outcomes improved, i.e., self-management activities and well-being increased while diabetes-related emotional distress and body weight decreased.

The observed effects on HbA1c are comparable with another study applying the same study design to test the preliminary effectiveness of the digital lifestyle intervention app Vitadio and reporting an average HbA1c reduction for the intervention group of −0.9% compared to 0.3% in the control period [16]. A similar study design was used to analyze a more interaction with healthcare providers-centered app-based intervention for T2DM patients (Vida Health App), which also showed a significant decrease in the 3-month intervention period (−0.8%) while HbA1c levels increased in the control period (+0.4%) [25]. However, the assessment time points were less tightly defined, and pre-enrolment HbA1c values were assessed up to 12 months prior to program enrolment [25]. 

Other 3-month trials that analyze app-based interventions for the treatment of T2DM patients with an intervention and control group mostly show significantly higher reductions in HbA1c levels compared to the control groups as well (between −0.5% and −1.3%) [26,27]. 

In a recent meta-analysis looking at telemedicine, higher baseline HbA1c, younger age (<55 years), and shorter duration of diabetes disease were also associated with increased benefits [13]. In the current trial, as well as the comparable Vitadio trial, only higher baseline HbA1c values yielded significantly higher reductions [16]. Other possible confounders like age, gender, or baseline BMI did not yield significance. 

Confounders that can also impact glycemic control like the duration of DMT2 or additional medication are rarely considered in analyses due to their complexity. In the current trial, the medication was assessed; changes were observed in both directions, i.e., reductions as well as increases in the number as well as doses. 

The impact of the baseline HbA1c values reflected in the analysis of the influence of app components on HbA1c development seems to be the most relevant factor in improving glycemic control. A study also predefining app engagement by the completion of level did not find differences in HbA1c values between (non-)completers of all levels, either [28]. Yet, the current study shows that the average available tracked time of physical activity is fulfilling current guidelines of at least 150 min of moderate to vigorous per week [8]. However, in not reflecting development over time and not considering patients who did not provide tracking data, the available app-generated data are limited. Studies taking into consideration the different features of the app and the actual input over time show that patients develop healthier diets or that tracking regularity of physiological health parameters and sticking to (individual) set goals positively influence health outcomes like HbA1c values or weight [16,29]. Future studies with larger sample sizes and more specified usage data might help generate more reliable insights.

### Strengths and Limitations

Except for technical aspects, study personnel did not interfere with patients evaluating the mebix under largely real-life conditions and obtained realistic evidence. Being recruited online could have caused a selection bias. Particularly patients who are interested in digital interventions and are generally more motivated to improve their glycemic control might have been included in the study. Since patients were free to use the app in accordance with their own time availability and liking, it was not possible to control the regularity and extent of their app usage.

One of the main limitations of most studies on digital health applications is the lack of blinding and a placebo control [12], which is also the case for the current study. However, due to the design with a retrospective control group, the values of the control period are not influenced by knowledge of participating in a study. This design also causes several missing (retrospectively assessed) HbA1c values. However, in substituting missing values with mean values, no intervention time was favored by data imputation.

Concerning the PROMs, no values for the control period are available and the number of missing values is high. While the results gave first insights into the potential positive impact of mebix on well-being and self-management, they did not yield significance and will have to be investigated further in future trials.

To improve the validity and generalizability as well as overcome some of the mentioned limitations, an RCT with an extended sample size and a parallel control group using a placebo app is planned for further evaluation of mebix. Pilot studies offer a fast and cost-effective generation of preliminary evidence for DiGAs, decreasing obstacles in the development of free digital therapies and also enabling smaller companies to enter the digital therapy market.

## 5. Conclusions

The present study demonstrates that the app-based intervention, mebix, may be effective in lowering HbA1c and reducing weight in patients with T2DM after 3 months. Moreover, the first data show the potential to improve well-being and self-management. Further research is needed to confirm the results and understand how the different contents and functions of the app affect diabetes therapy.

## Figures and Tables

**Table 1:** Effects on glycemic control and metabolic parameters.

	Retrospective	Baseline	Follow-Up	Change	*p*-Value
	(−3 Months)		(+3 Months)		
n = 48					
HbA1c (%) Control group	8.4 ± 0.9	8.3 ± 0.7	-	−0.2 ± 0.8	0.177
**HbA1c (%)** Intervention group	-	8.3 ± 0.7	7.3 ± 0.6	−1.0 ± 0.8	<0.001 ***
n = 39					
Weight (kg)	-	101.2 ± 18.1	98.2 ± 17.7	−2.9 ± 3.4 kg	<0.001 ***
BMI (kg/m^2^)	-	35 ± 6.5	34 ± 6.5	1 ± 1.1	<0.001 ***

Note: Signif. codes: 0 ‘***’ 0.001 ‘.’ 0.1 ‘ ’ 1.

**Table 2:** Patient-reported outcomes.

PRO	Baseline	Follow-Up (+3 Months)	*p*-Value
	N = 37 ^1^	N = 28 ^1^	N = 26 ^1^
**WHO-5**	13.0 ± 6.0	14.5 ± 6.0	0.163
**PAID-5**	7.16 ± 5.2	6.68 ± 4.6	0.120
**SDSCA**			
*Overall Scale*	3.38 ± 1.2	3.99 ± 1.2	0.120
*Diet*	4.03 ± 1.3	4.47 ± 1.2	1.000
*Exercise*	3.41 ± 1.5	3.66 ± 1.8	1.000
*Blood Glucose Testing*	4.12 ± 2.8	4.54 ± 2.7	1.000
*Footcare*	1.32 ± 1.9	2.82 ± 2.2	<0.001 ***

Note: PAID-5, Problem Areas in Diabetes questionnaire; SDSCA, Summary of Diabetes Self-Care Activities measure; WHO-5, WHO-Five Well-Being Index. The *p*-values of the SDSCA overall and subscales are corrected *p*-values with the Bonferroni correction. Signif. codes: 0 ‘***’ 0.001 ‘.’ 0.1 ‘ ’ 1. ^1^ The different number of participants at baseline, follow-up and included in the analysis is because both the baseline and the follow-up questionnaire are only available for 26 participants.

**Table 3:** Analysis of Variance Table of the linear model to predict HbA1c changes.

Predictor	β	df	Sum of Squares	F Value	*p* Value
Average daily steps	0.000	1	3.0364	13.4482	0.006 **
Average weekly physical activity (in minutes)	−0.001	1	0.0465	0.2058	0.662
All 12 weekly tasks completed (yes/no)	−1.371	1	0.4286	1.8981	0.206
At least 3 food logs (yes/no)	0.640	1	3.4038	15.0753	0.005 **
Baseline HbA1c (in %)	−1.316	1	8.0071	35.4634	0.000 ***
Residuals		8	1.8063		

Note: β, Coefficient; df, degrees of freedom. Signif. codes: 0 ‘***’ 0.001 ‘**’ 0.01 ‘.’ 0.1 ‘ ’ 1.

## Data Availability

The data presented in this study are available to researchers who submit a methodologically sound proposal to the principal investigator, P.E.H.S. (peter.schwarz@ukdd.de). To gain access to the data, proposers will be required to sign a data access agreement.

## Supplementary Materials

The following supporting information can be downloaded at: https://www.mdpi.com/article/10.3390/jcm12196317/s1, Figure S1: Home screen with presonal goals, activity, and water tracking; Figure S2: Weekly plan with personal (daily) activity goals and tracking; Figure S3. Step count overview; Figure S4. Example for educational video content.
