# Supplementary material for: Effectiveness of a Digital Health Application for the Treatment of Diabetes Type II—A Pilot Study
Source: J Clin Med. 2023 Sep 30;12(19):6317. doi: 10.3390/jcm12196317 (PMC10573202; doi:10.3390/jcm12196317)
Supplement: Supplementary file 1 [file jcm-12-06317-s001.zip › jcm-2631955-supplementary.pdf]

Screenshots of the App

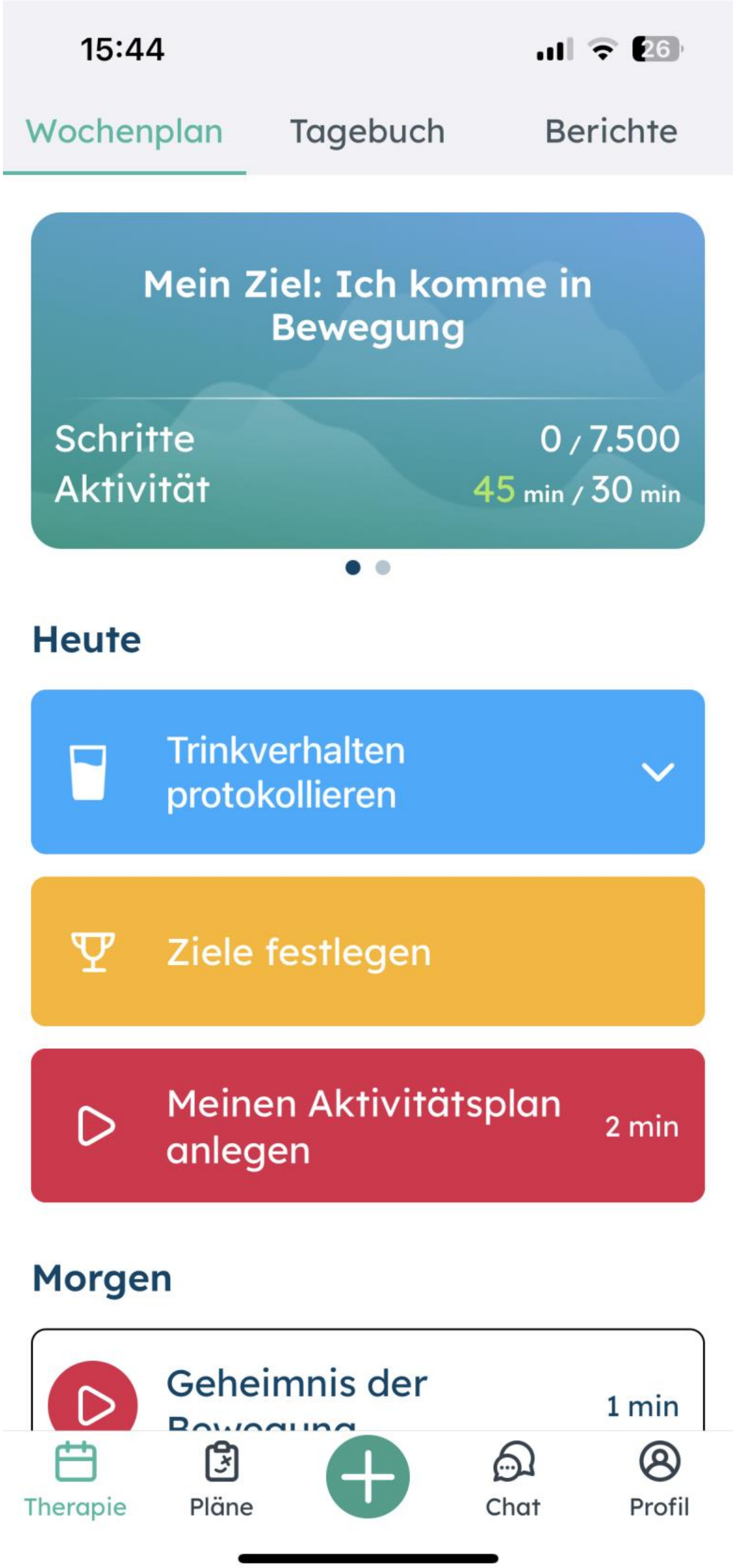

Figure S1. Home screen with presonal goals, activity, and water tracking.

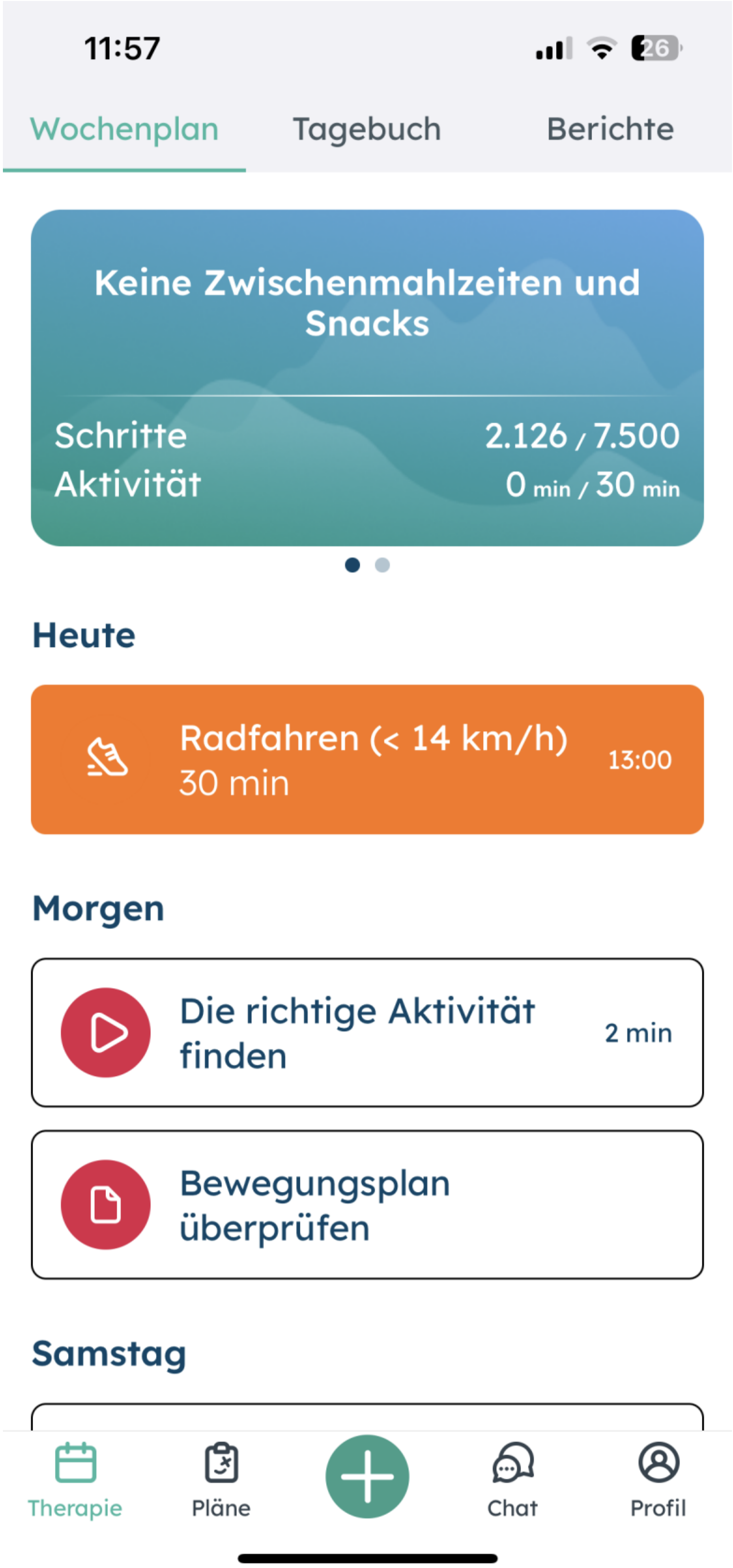

Figure S2. Weekly plan with personal (daily) activity goals and tracking.

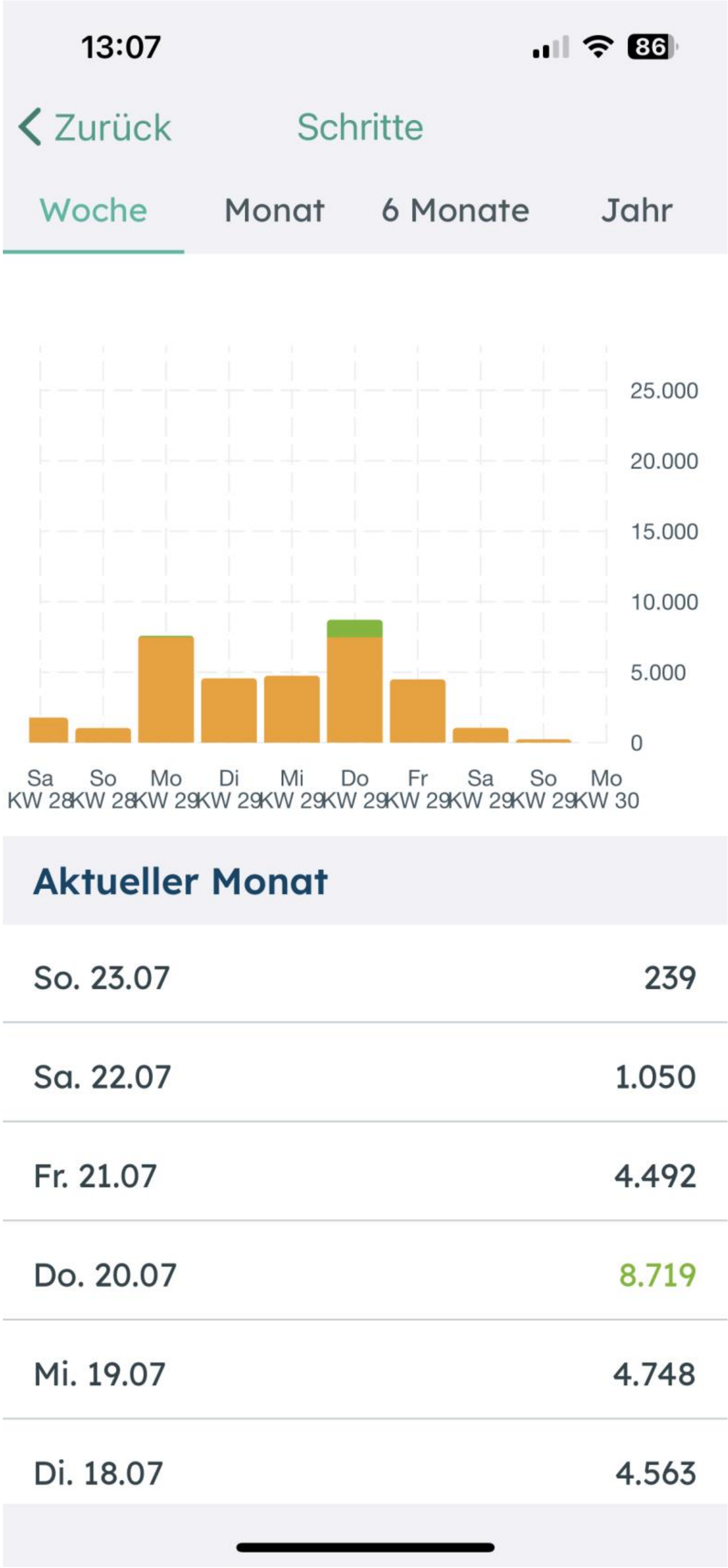

Figure S3. Step count overview.

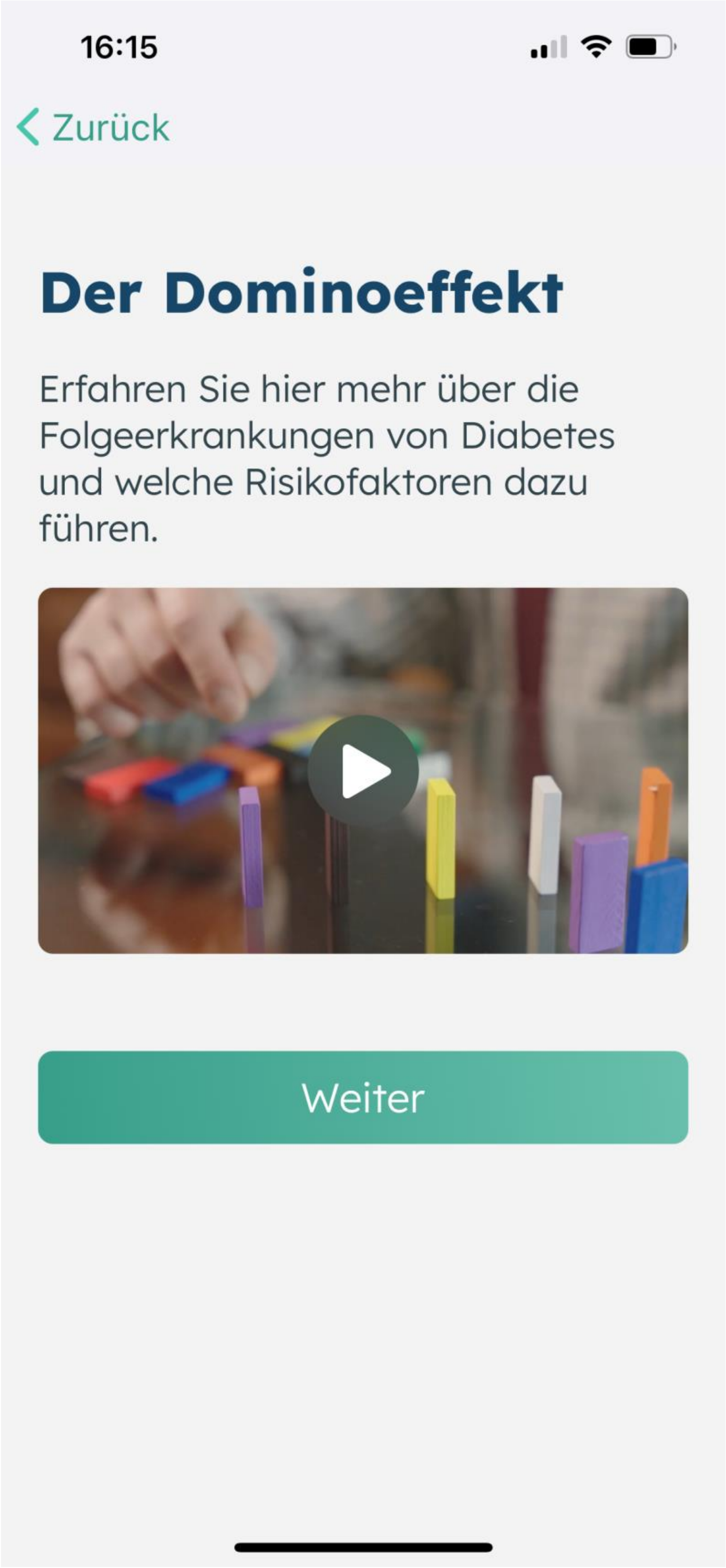

Figure S4. Example for educational video content.
